# The transcendent voice of recovery mentors in mental health: a philosophical reflection

**DOI:** 10.3934/publichealth.2019.2.135

**Published:** 2019-04-15

**Authors:** Jean-François Pelletier

**Affiliations:** 1 Department of Psychiatry and Addictology, University of Montreal, Canada; 2 Department of Psychiatry, Yale University School of Medicine, USA

**Keywords:** global health, recovery mentorship, peer support, hermeneutic, whole-person care

## Abstract

In a globalized world health and illness know no frontiers. Pandemics have never been limited to political borders and the contemporary campaigns to prevent them can be effective only when addressed not only internationally but also with the application of integrated disease management in order to respond to problems caused by the silo approach. In any case, it appears that global health has been constantly in redefinition. With this commentary a phenomenological redefinition of global health is proposed as an integrative strategy. Phenomenology prioritizes and investigates from the first-person point of view how the human being experiences the world, as it explores the unique meaning of the lived experience of being human. We are particularly interested in verifying if and how, from a first-person point of view, the lived experience of mental illness and of recovery can contribute to a more integrated definition of global health. In the field of mental health, formal peer support is a mentor/mentee relationship, and as such it is an emotional and practical support between two people who share a common experience of a mental health challenge or illness. Peer support is a system of giving and receiving help founded on key principles of respect, shared responsibility, and mutual agreement of what is helpful. It is about understanding another's situation empathically through the shared experience of emotional and psychological pain. And when speaking in public, a recovery mentor accepts to disclose and to be recognized as a (former) mental health service user. That person knows that there is a possibility of being stigmatized, but yet remains courageously engaged towards the promotion of change and in solidarity with people who are suffering worldwide and who may not have this opportunity to speak freely.

## 1. Introduction

In 2009, Koplan and colleagues defined global health as “the area of study, research and practice that places a priority on improving health and achieving equity in health for all people worldwide” [1]. Indeed, in a globalized world health and illness know no frontiers. Firstly conceived from a geopolitical and international public health perspective, global health is often thought of as a strategy to help low- and middle-income countries of the South to scale up towards Northern standards for better access to quality treatments that have been proven effective in high-income countries of the North. But nowadays, especially with climate change as a major threat to global health [2], even in the high-income countries it is more obvious than ever that any public health issue needs to be thought of not only as national, but also in terms of its possible multidirectional implications, causes, or solutions that could be found outside of the country's borders. Pandemics have never been limited to political boundaries and the contemporary campaigns to prevent the propagation of AIDS or Ebola, just to take these examples, can be effective only when addressed not only internationally but also with the application of integrated disease management in order to respond to problems caused by the silo approach [3].

Global public health is intimately linked with political, economic and social determinants [4], all of which should be addressed in an integrated manner. Yet, although the call for integration is common in policy discussions of global health, the definition of integration has not always been clear [5]. There is a risk that the term “global health” would therefore remain only a vague umbrella term [6]. Furthermore, in recent years, the advent of the internet and social media have dramatically changed access to medical information and modified the sense of belonging and of affiliation between individuals and ethno-cultural groups around the world. Communities are less and less solely defined geographically and the classic geopolitical definition of global health must therefore be reviewed in the light of these macro- and micro-societal evolutions as well.

In any case, it appears that global health has been constantly in redefinition. This might be a sign of wisdom rather than of conceptual weakness because the world, along with health issues and technologies, is also constantly changing, and apparently at a faster pace than ever. As well put by João and Petryna [7], simply engaging with the complexity of people's lives—their constraints, resources, subjectivities, projects—in unfixed and multilayered social worlds requires the constant resetting of conceptual lenses and standards of evidence-making.

With this commentary a phenomenological redefinition of global health is proposed as an integrative strategy. Phenomenology prioritizes and investigates from the first-person point of view how the human being experiences the world, as it explores the unique meaning of the lived experience of being human. In the following, we are particularly interested in verifying if and how, from a first-person point of view, the lived experience of recovery in mental health and the sharing of this experience can contribute to a more integrated definition of global health.

## 2. Crossing the axes

In matters of access to efficient and evidence-based treatments, for communicable and for non-communicable diseases as well, academics in high-income countries are now expected to be more cognizant of social iniquities and disparities faced by low- and middle-income countries of the rest of the world. The contemporary migratory flows add to this complexity by importing in the former some of the problems typical to the latter. This is one of the reasons why, within health professional education in universities around the globe, there now exists a growing awareness of the professional duty to be socially accountable. This implies being attentive to the needs of all members of communities, regions, and nations, especially those who disproportionately suffer from the adverse influence of social determinants [8]. This is the case for people with lived experience of mental illness, or more precisely for people with lived experience of having been labelled as mentally ill. Although the social determinants of physical health and those of mental health are largely the same, the negative social representations of mental illness and of psychiatric disorders are an additional barrier for accessing quality care because people who suffer from these very often simply do not want to seek care and services of this nature. They have fear of being stigmatized if they are seen going to or coming back from psychiatric institutions. From the point of view of a patient who lives with combined mental and physical chronic health conditions, which is often the case, the dichotomy between mental health and physical health is an artificial frontier to be overcome for a truly global approach to health and overall wellbeing. This is even more so when this dichotomy is architecturally inscribed by the construction and maintenance of clearly distinguishable buildings: some for physical health and some others for mental health. Patients with chronic co-morbidities who are being monitored in primary care are not alternatively part-time mentally ill and part-time physically ill; their mental and physical conditions are continuously interwoven.

After a first geopolitical axis of global health where the territorial borders are those to the sovereignty of the nation states, a second axis for considering health globally would be to pay particular attention to the entanglement of the physical, mental, and social dimensions of health instead of treating them, as usual, too separately and sometimes even as far as possible from one another. Although the World Health Organization (WHO) has defined health itself as *a state of complete physical, mental and social well-being and not merely the absence of disease or infirmity*, mental health systems and physical health systems have historically evolved in silos in Western civilization. This was done to the detriment of mental health with an extra burden due to stigma against the mentally ill, discrimination, and overall lack of awareness about mental health. Thus the call from WHO to challenge the existing inhumane form of segregated institutional care services and not to replicate this duality in low- and middle-income countries but rather to foster better integration of real post-asylum mental health-care delivery directly in community-based primary care [9].

Mental disorders are common worldwide, yet the quality of care for these disorders has not increased to the same extent as that for physical conditions [10]. The disparity between the resources devoted to the treatments of mental illnesses and those of physical illnesses is well documented. The global burden of neuropsychiatric diseases and related mental health conditions is indeed underappreciated and under resourced, particularly in the developing nations [11]. Targeting this physical/mental iniquity would then make global health somehow more holistic [12] and would pave the way to whole person care. For example, there may be concerns about the poor physical health and greatly reduced life expectancy of people living with serious mental illness such as schizophrenia. These people generally die from the same complications of chronic physical illnesses as the rest of the population does, but much more prematurely, that is up to 20 years sooner, or even more when the combined effects of social determinants that disadvantage them are taken into account (i.e. when socio-demographics are not controlled for when life expectancy with schizophrenia is discussed). In the other direction, people living with incurable chronic diseases or with cancer are prone to also suffer from common mental disorders such as anxiety or depression, which negatively impact their prognosis by weakening their potential for resiliency and self-regulation, their understanding, and their adherence to treatments. As some chronic physical illnesses can be at least partially prevented through self-regulation and changing one's lifestyle and behaviors [13], regular physical activity is also well known to provide a range of both physical and mental health benefits [14]. Although complementary to one another, the promotional approach to health and exercise on one hand, and the curative approach to illness on the other hand, are not the same. For a particular individual or for a population as a whole, a combination of these two approaches may also characterize a more holistic definition for global health.

This second holistic axis of globalism can reinforce the abovementioned first geopolitical axis. The promulgation of national and international public health policies covering the promotional approach and the curative approach can be done by jointly addressing the social determinants of mental and physical health, be they endogenous or exogenous to a precise jurisdiction, and with an integrated intersectional approach of multilateral cooperation.

## 3. Body and mind

The WHO estimates that one in four people in the world will be affected by mental or neurological disorders at some point in their lives. In 2001 around 450 million people were suffering personally from such conditions, placing mental disorders among the leading causes of ill-health and disability worldwide [15]. Since neuropsychiatric disorders account for more than 10% of the global burden of disease, the American Psychiatric Association recommends a global mental health approach to medical education that touches on various topics including developing cultural competencies, understanding epidemiology in cultural contexts, and finding ways to improve access to care and treatment [16]. However, there is still a tension between a vertical top-down approach to public mental health, based on a biomedical framework that applies to all human beings (with neuropsychiatric disorders being presumably biologically determined and universal), and a more horizontal community-based and bottom-up approach that emphasizes empowerment of local resources and endogenous solutions [17]. Global health is about the reduction of disparities and protection of societies against global threats that disregard national ethno-cultural borders and communitarian integration. To counterbalance the top-down Western bio-medical hegemony, academic institutions are invited to reach across geographic, cultural, economic, gender, and linguistic boundaries to develop a mutual understanding of the scope of global health and to create collaborative education programs [18]. Western standard treatments cannot be readily applied across cultures with just minimal adaptation. Indigenous forms of healing and coping may also contribute to positive outcomes and recovery when respectfully acknowledged. Transcultural psychiatry practitioners know very well that it is in fact counterproductive to be content with proposing treatments that are at odds with the cultural benchmarks in which an individual normally evolves on a daily basis, even though the treating team is familiar with it for having been trained to prescribe it. This is not a matter of absolute relativism of antipsychiatry, but more of centering on clinical facts and helping clinicians in their primary task of alleviating suffering [19]. Undoubtedly, providing care that matches one's cultural needs is an essential component of effective and responsive mental health care delivery. Yet, the mainstream development of the psychiatric knowledge is still mostly influenced by the positivist research paradigm. As with nursing [20], there is a need for psychiatry to address the utility of human sciences or the humanistic philosophy that values the understanding of subjective experiences.

Indeed, studies on the relationship between thought and affectivity, on theories of motivation and on various clinical syndromes show how the acquisition of increasingly complex knowledge on the central nervous system requires a change in the human's conception to take into account his subjectivity. Medical clinical sciences are gradually rediscovering the importance of considering this subjectivity, starting with the individual patient's story in its sociocultural context and not limiting the scope to positivist epistemological principles that too narrowly stress the biological processes of the disease; hence the need for *narrative medicine*. This attention to the subject as a whole rather than to its different systems or organs opens up to the taking into account of subjectivity. As phenomenology seeks to discern and to understand the lived experience from the inside, this experience needs first to be heard.

A third way to define global health would thus be to do it epistemologically. In Western civilization two broad classes of knowledge about the human experience exist while sometimes ignoring each other: that of the body and that of the mind. These epistemologies are often presented as dialectical and dualistic, if not historically antagonistic to one another when in competition for public resources allocation. These two orientations are nevertheless equally as indispensable to medicine because most of the differences between individuals, families, and communities are decidedly, for the most part, not physiological. To understand and alleviate the suffering of his or her patient, a physician must interpret subjectively the narrative and the behavior of that person. This interpretation can only be done in reference to the social norms and representations of the society to which both the patient and the physician belong, though they may not belong to the same social class. This is why “Medical humanities” are now increasingly present in American medical schools. Similarly, in recent decades, several European countries have incorporated social science concepts into medical education [21].

## 4. The integrative voice of recovery mentors

Mentor is a character of the *Odyssey*, which is a major ancient Greek epic poem attributed to Homer. Known as a wise adviser and sage counselor, Mentor provided guidance, encouragement and practical plans to Ulysses's son, Telemachus, for dealing with personal dilemmas. The name Mentor has been adopted in English as a term meaning someone who inspires wisdom and translates knowledge. Mentorship is thus the developmental relationship between a more experienced mentor and a less experienced “mentee”. The mentor's role is to listen, provide constructive feedback, help the mentee consider various options, refer them to resources available and facilitate their decision-making [22]. In the field of mental health, formal peer support is a mentor/mentee relationship, and as such it is an emotional and practical support between two people who share a common experience of a mental health challenge or illness. As defined by Shery Mead, peer support is a system of giving and receiving help founded on key principles of respect, shared responsibility, and mutual agreement of what is helpful. Peer support is about understanding another's situation empathically through the shared experience of emotional and psychological pain. This affiliation is a deep, holistic understanding based on mutual experience where people are able to “be” with each other without the constraints of traditional expert/patient relationships [23]. It is thus much more horizontal than the typical expert- or doctor-patient relation because recovery mentors have lived through that similar experience, and are trained to support others by sharing with parsimony their own experience on an equal basis rather than from an expert standpoint. They adopt a position so that the mentees, from whom the mentors can learn too, can recognize themselves in the mentors as vis-à-vis. It is this mirror relationship of reciprocity that allows the emulation of recovery. Tracy and colleagues [24] provide a clinical example of the role of mentors in a training intervention for alcohol abusers, namely the *Mentorship for Alcohol Problems* intervention. Based on social learning theory, the mentor helps the mentee to develop and achieve abstinence goals using harm reduction strategies which are monitored through modified goal attainment scaling. Positive results to fidelity measures of this pilot study suggest that this *Mentorship for Alcohol Problems* intervention formalizes client-to-client mentorship relationships as an adjunct to standard outpatient treatment. This effect is partly due to the fact that mentors can provide a continuing supportive relationship outside of the treatment setting.

Then, when speaking in public, a recovery mentor accepts to disclose and to be recognized as a (former) mental health service user. That person knows that there is a possibility of being stigmatized, but yet remains courageously engaged towards the promotion of change and in solidarity with people who are suffering worldwide and who may not have this opportunity to speak freely and self-emancipated from any label.

Peer support is now considered to be a central component of the behavioral health care system in countries such as the US, Canada, Australia and the UK [25]. It might also be most relevant in low-resource countries and non-Western cultures. Indeed, besides the usual discursive knowledge and methods of traditional evidence-based medical science, attention must also be paid to the subjective knowledge to reflect the depth of human experience [26]. As recommended since 2013 by *BMJ Case Reports*, the global health problem analysis should examine the problems of individual patients and describe actual and potential solutions for the patient, the local community, and patients affected by similar issues across the world [27]. The added value of the recovery mentor expertise is to provide a patient focus and real-life context in the hermeneutical analysis of global health problems that includes and values its subjectivity and experiential knowledge.

Typically, peer support can happen in both group and one-to-one relationships. In either scenario, the recovery mentor provides emotional and social support to others who share a common experience. At the international level, they can advocate for more inclusive public health policies and interventions. Being able to refer to international conventions and human rights standards is a key component of a genuine global approach that is supportive of individuals and communities in their quest for recovery.

Published for the first time in 2009 and revised in 2013 [28],[29], the Global Model of Public Mental Health was first inspired by the ecological approach in public health and health promotion programs, while adding to that approach the recovery mentors as emulators of personal transformation and agents of mental health policies and legislation transformation. This approach to agency is said to be global in that the supranational and individual levels reinforce each other, taking turns with a) a set of legal rules and international conventions on human rights, including those of disabled persons and the WHO's recovery-oriented Mental Health Action Plan 2013–2020 [30], and b) the active involvement of engaged patient advocates and recovery mentors who can evoke these rules and conventions as part of a plea for the recognition of their personal and collective capacity for change. Local communities and persons in recovery can reach each other to promote change and capacity building, for instance through quality assessment, and evaluation of human rights' level of respect in healthcare facilities and more broadly. Recovery mentors can thus reveal obstacles and gateways in the recovery journey which by essence, is intersectional. As spokespersons recovery mentors can alternately speak using the “we” or express themselves using the “I.” In either case, using their lived experience of recovery, their role as spokespersons is multi-directional. In one direction, the recovery mentors can inform the governmental and institutional stakeholders about the expectations of typical patients and about their daily life reality of going through social determinants because they have lived it and reflected on it. In the other way, their role is to explain and make more intelligible for their mentees the intricacies of the system, with a lay language, while instilling hope for a better future.

Is it possible to develop a conceptual synergy between global health defined 1) geographically and in terms of social responsibility, 2) holistically in terms of complementarity of physical, mental and social health, and 3) epistemologically in terms of scientific reconciliation of subjectivity and objectivity? A fourth hermeneutical axis, transversal to those other three, would be to promote the wisdom of recovery mentors by providing them opportunities to speak out freely. For example, this can be done by inviting recovery mentors and recovery mentees to share their lived experience and experiential knowledge in order to inform and educate health professionals, and other stakeholders, of the existence of this wonderful opportunity for recovery in global health [31].
